# Neural processing of vision and language in kindergarten is associated with prereading skills and predicts future literacy

**DOI:** 10.1002/hbm.25449

**Published:** 2021-05-04

**Authors:** Johanna Liebig, Eva Froehlich, Teresa Sylvester, Mario Braun, Hauke R. Heekeren, Johannes C. Ziegler, Arthur M. Jacobs

**Affiliations:** ^1^ Department of Education and Psychology Freie Universität Berlin Berlin Germany; ^2^ Center for Cognitive Neuroscience Berlin Freie Universität Berlin Berlin Germany; ^3^ Centre for Cognitive Neuroscience Universität Salzburg Salzburg Austria; ^4^ Deparment of Biological Psychology and Cognitive Neuroscience Freie Universität Berlin Berlin Germany; ^5^ Aix‐Marseille Université and Centre National de la Recherche Scientifique, Laboratoire de Psychologie Cognitive Marseille France; ^6^ Present address: Department of Decision Neuroscience and Nutrition German Institute of Human Nutrition Potsdam‐Rehbrücke Nuthetal Germany

**Keywords:** child, fmri, longitudinal, reading

## Abstract

The main objective of this longitudinal study was to investigate the neural predictors of reading acquisition. For this purpose, we followed a sample of 54 children from the end of kindergarten to the end of second grade. Preliterate children were tested for visual symbol (checkerboards, houses, faces, written words) and auditory language processing (spoken words) using a passive functional magnetic resonance imaging paradigm. To examine brain–behavior relationships, we also tested cognitive–linguistic prereading skills at kindergarten age and reading performance of 48 of the same children 2 years later. Face‐selective response in the bilateral fusiform gyrus was positively associated with rapid automatized naming (RAN). Response to both spoken and written words at preliterate age was negatively associated with RAN in the dorsal temporo‐parietal language system. Longitudinally, neural response to faces in the ventral stream predicted future reading fluency. Here, stronger neural activity in inferior and middle temporal gyri at kindergarten age was associated with higher reading performance. Our results suggest that interindividual differences in the neural system of language and reading affect literacy acquisition and thus might serve as a marker for successful reading acquisition in preliterate children.

## Graphical abstract

Longitudinal study examining reading acquisition from preliterate kindergarten age to the end of the second year of primary school. We used a passive fMRI paradigm to study the brain–behavior relationships of vision, language and reading.

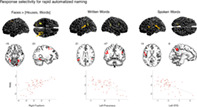

## 1 INTRODUCTION

When children learn to read, they are confronted with a highly complex and challenging task in which they have to map a novel visual symbol system onto partially pre‐existing spoken language representations (Ziegler, Perry, & Zorzi, 2020). Consequently, the visual and spoken language system and neural pathways that link them undergo major structural and functional changes during development (Dehaene et al., 2010; Dehaene, Cohen, Morais, & Kolinsky, 2015; Monzalvo & Dehaene‐Lambertz, 2013). Despite general changes that can be seen in every literate brain (e.g., emergence of the visual word form area, VWFA), there seem to be fine interindividual differences in the neurofunctional anatomy of regions that later become part of the reading network and beyond (Skeide et al., 2017). These differences might affect literacy acquisition and, in some cases, might result in developmental disorders, such as dyslexia (see Perry, Zorzi, & Ziegler, 2019, for a computational approach). The behavioral cognitive–linguistic skills associated with literacy development are well described (e.g., Landerl et al., 2013, 2019; Scarborough, 1998). For instance, children are known to become sensitive to phonological units smaller than the whole word, which is an important causal predictor of reading proficiency in later developmental stages (Goswami, 2000; Hulme & Snowling, 2013; Melby‐Lervåg, Lyster, & Hulme, 2012; Ziegler et al., 2010; Ziegler & Goswami, 2005). Comparatively, less research is available on the causal role of visual factors (Goswami, 2015) and even fewer studies have examined the neural predictors of literacy development (Ozernov‐Palchik & Gaab, 2016; Vandermosten, Hoeft, & Norton, 2016 for a meta‐analysis and review). It is still a controversial and open research question as to whether specific preliterate neural correlates can reliably predict future literacy. An answer to this question would be extremely useful for the identification of children who are at risk of dyslexia.

### 1.1 Neural systems for vision, spoken and written language

In adults, the visual stream located in the ventral occipito‐temporal (vOT) cortex has a highly systematic spatial and functional organization encompassing selective subregions specifically tuned to recognize certain behaviorally‐relevant object categories (Malach, Levy, & Hasson, 2002). The ventral cortex of infants has recently been found to broadly show the same functional division as the one seen in adults (Deen et al., 2017). Conversely, however, the category specificity, as attributed to the vOT, is not yet mature in infants, and undergoes substantial changes. Response selectivity to faces, for instance, increases until adulthood, highlighting the plasticity of the ventral stream (Deen et al., 2017; Dehaene & Cohen, 2007; Gomez et al., 2017). Of special interest in the present study is the rapid emergence of print sensitivity in the fusiform gyrus during reading acquisition (Brem et al., 2010; Chyl et al., 2018; Dehaene‐Lambertz, Monzalvo, & Dehaene, 2018; Monzalvo, Fluss, Billard, Dehaene, & Dehaene‐Lambertz, 2012; Seghier, Lee, Schofield, Ellis, & Price, 2008). In contrast to earlier proposals (Dehaene et al., 2010), reading acquisition does not seem to conquer face‐selective visual regions but rather uncommitted neurons that become responsive to print during the first steps of reading acquisition (Dehaene‐Lambertz et al., 2018). Nonetheless, face processing seems to partly shift to the right hemisphere with increasing exposure to print (Dehaene‐Lambertz et al., 2018) and the ventral stream becomes the host for fast whole word processing (Grainger, Lété, Bertand, Dufau, & Ziegler, 2012; Grainger & Ziegler, 2011; Seghier et al., 2008).

Auditory language comprehension, meanwhile, starts with prelexical phonemic processing in the auditory cortex (Heschel's gyrus) and then progresses towards the bilateral superior temporal gyrus (STG) and sulcus (STS) (Hickok & Poeppel, 2007; Price, 2010). Semantic retrieval is additionally associated with a distributed network encompassing, among others, the supramarginal gyrus (SMG) and angular gyrus (AG) (Binder, Desai, Graves, & Conant, 2009; Price, 2010). The dorsal reading stream, which largely relies on this left‐hemispheric temporo‐parietal (TP) language network, is associated with sublexical reading, that is, grapheme‐to‐phoneme mapping (Boros et al., 2016; Braun et al., 2015; Vinckier et al., 2007). In skilled readers, the bilateral planum temporale , STG (Richlan, 2019), and particularly the posterior dorsal STS (Wilson, Bautista, & McCarron, 2018), have been identified as important convergence zones for letter speech sound integration.

In children beginning to learn to read, the TP language network needs to be fundamentally reorganized in order to become responsive to print (Brem et al., 2010; Frost et al., 2009; Montant, Schön, Anton, & Ziegler, 2011; Preston et al., 2016). In fact, during reading acquisition, audio‐visual language integration modulates not only responses in the dorsal system, that is, the STS and STG, but also in the ventral stream including the fusiform gyrus. This was recently shown in an artificial letter‐learning paradigm mimicking the first steps of reading acquisition in prereaders (Karipidis et al., 2017; Karipidis et al., 2018; Pleisch et al., 2019; Plewko et al., 2018). The results of their letter‐learning paradigm impressively show that the ability to learn grapheme‐to‐phoneme correspondences is a key factor in reorganizing the dorsal and ventral stream to become responsive to print, which forms an essential prerequisite for their rapidly emerging interdependence (Liebig et al., 2017).

### 1.2 Predictors of literacy

At the behavioral level, several studies have shown that cognitive–linguistic prereading skills measured prior to reading instruction, such as rapid auditory processing (Pugh et al., 2013; van Zuijen, Plakas, Maassen, Maurits, & van der Leij, 2013), visual and auditory attention (Franceschini, Gori, Ruffino, Pedrolli, & Facoetti, 2012; Lallier, Thierry, & Tainturier, 2013), or visual motion perception (Boets, Vandermosten, Cornelissen, Wouters, & Ghesquière, 2011; Gori, Seitz, Ronconi, Franceschini, & Facoetti, 2016) predict later reading skills (Lervåg, Hulme, & Melby‐Lervåg, 2018). In the present study, we focus on phonological awareness (PA) and rapid automatized naming (RAN) as cardinal behavioral predictors of literacy. Their strong relationship has been reliably shown in multiple large‐scale cross‐linguistic studies at a concurrent (Landerl et al., 2013; Moll et al., 2014) as well as a longitudinal level (Caravolas et al., 2012; Landerl et al., 2019; van Bergen et al., 2011).

At the neural level, a number of neuroimaging studies have shown differences in the reading network of children depending on their level of reading proficiency (e.g., Ben‐Shachar, Dougherty, Deutsch, & Wandell, 2011; Turkeltaub, Gareau, Flowers, Zeffiro, & Eden, 2003). To gain a deeper knowledge of the trajectories of reading development and to better understand why some children fail to become efficient readers (Hoeft et al., 2007; Perry et al., 2019), there is an increasing effort to study structural (e.g., Kraft et al., 2015) and functional (e.g., Hong et al., 2018; Liebig, Friederici, & Neef, 2020; Lohvansuu, Hämäläinen, Ervast, Lyytinen, & Leppänen, 2018; Morken, Helland, Hugdahl, & Specht, 2017; Neef et al., 2017) neural predictors of future reading proficiency before the onset of literacy. On the neurophysiological level, a strong link between basic auditory processing and later reading development has been reported in kindergarten children (for a review see Hämäläinen, Salminen, & Leppänen, 2013). Longitudinal structural magnetic resonance imaging starting at preliterate age suggests anatomical differences in reading‐related regions associated with cognitive–linguistic prereading skills (Beelen, Vanderauwera, Wouters, Vandermosten, & Ghesquière, 2019; Raschle, Chang, & Gaab, 2011). More specifically, Raschle et al. (2011) reported a positive correlation between the gray matter volume in the left vOT, planum temporale, and RAN. Similarly, Beelen et al. (2019) showed that the surface area and cortical thickness of the left fusiform gyrus positively correlates with PA. Moreover, the observed brain–behavior relationships are not confined to structural indices.

Functional neuroimaging (fMRI) showed neural activity in the ventral stream in response to letters prior to reading instruction. Preliterate response to print in the fusiform gyrus not only predicted word reading at the end of the second grade (Centanni et al., 2018), but also differed significantly in children who struggled with reading acquisition (Centanni et al., 2019). Similarly, Karipidis et al. (2018) showed that audio‐visual integration of print in the left ventral and dorsal stream was positively associated with future reading fluency (5–7 month of reading instruction). Likewise, activation strength in the preliterate auditory language network was strongly related to subsequent reading performance in beginning (shortly after school enrolment) and emergent readers (two  years of instruction; Yu et al., 2018). Moreover, spoken language ability in emergent readers seems to shape print‐speech convergence in the bilateral inferior frontal gyrus (IFG), STG, middle temporal gyrus (MTG), and parietal regions (among others), and interindividual differences in the print‐speech coactivation strength in the MTG and STG has been shown to predict reading performance 1 year later (Marks et al., 2019). These longitudinal results demonstrate that the sensitivity of key regions of the future ventral and dorsal reading stream systematically differ before the onset of literacy acquisition. Chyl et al. (2018) used a passive fMRI paradigm to compare the neural systems of written and spoken language in kindergarten children and beginning readers. For the reading cohort, they reported that the neural response to written words in the ventral stream (left inferior occipital and fusiform gyrus) and a widely distributed network of bilateral regions correlated with sight word reading, while print‐specific response negatively correlated with reading performance in bilateral frontal regions and left precuneus. Furthermore, activation in bilateral temporal regions (STG, MTG, among others) in response to speech was positively associated with reading skills, while neural response in the left inferior parietal lobe of the dorsal stream correlated negatively with reading. Dehaene‐Lambertz et al. (2018) used a simple target‐detection task to longitudinally examine the neural reorganization of vision and language throughout the first year of formal reading instruction. They found that reading fluency was associated with response selectivity to visual stimuli. Most of the studies mentioned above confined the analysis of neural correlates and predictors of reading to linguistic stimuli. In contrast, Dehaene‐Lambertz et al. (2018) included further visual categories, in particular faces. In fact, reading fluency correlated with increased activation in left vOT and the right cerebellum not only when processing words and numbers, but was also associated with enhanced response to faces in the right fusiform gyrus (Dehaene‐Lambertz et al., 2018). Similar results were obtained in an earlier study by Monzalvo et al. (2012) who investigated neural markers of reading in a cohort of older children with varying reading proficiency. Their results showed strong differences in the neural response of the fusiform gyrus and planum temporale to faces, written words and spoken language in children with and without dyslexia. Recently, Nordt et al. (2019) examined various linguistic and nonlinguistic visual categories in relation to reading development in three different age cohorts (5–9, 10–12, 22–28 years). In their study, however, only the selective neural response to words in the left vOT was positively linked to reading ability in children and adults. In all these studies (Chyl et al., 2018; Dehaene‐Lambertz et al., 2018; Monzalvo et al., 2012; Nordt et al., 2019) neural markers of reading and behavioral reading performance were measured concurrently. Hence, it is possible that the modulation of the neural response captured the consequences of different reading proficiencies rather than their origin. Thus, the question remains as to whether modulation of neural processing is a cause or consequence of differences in literacy skills.

### 1.3 Present study

In the present study, we examined whether the “readiness” or sensitivity of neural systems in response to vision and language is associated with cognitive–linguistic prereading skills and predicts future reading. To do so, we followed children from kindergarten until the end of the second year of primary school. Neural sensitivity to different categories of visual stimuli (checkerboards, houses, faces, written words) and auditory language processing (spoken words) was obtained in preliterate children using a passive fMRI paradigm. Our research questions were threefold: First, we wanted to characterize visual and auditory language systems (baseline contrast for each stimulus condition and target stimulus > all other conditions for visual conditions) in kindergarten children, which have previously been well described in adults and adolescents (e.g., Dehaene et al., 2015; Hickok & Poeppel, 2007; Price, 2010). We expected neural responses to the nonlinguistic visual stimuli would occur in the ventral stream, comprising the inferior occipital gyrus (IOG), the vOT, and, in particular, the fusiform gyrus (Cantlon, Pinel, Dehaene, & Pelphrey, 2011; Dehaene‐Lambertz et al., 2018). According to Chyl et al. (2018), who reported only small and unspecific response to print compared to symbol strings in prereading children, we expected little and possibly unspecific activation in the ventral stream for written words > (all other visual conditions), as the cohort of kindergarten children was not able to actually decode print (cf. Cantlon et al., 2011). The baseline contrast of written words might, however, yield activation in bilateral temporal regions as reported for the same group of prereaders (Chyl et al., 2018), while spoken words should reliably activate the dorsal auditory language system, that is, the bilateral STG, STS and possibly also the AG and SMG for lexico‐semantic processing of words (Monzalvo & Dehaene‐Lambertz, 2013; Price, 2010; Enge et al., 2020).

Second, we wanted to examine the neural underpinnings of well‐known cognitive–linguistic skills which promote reading acquisition, namely PA and RAN, that are systematically associated with successful reading acquisition in different writing systems (Landerl et al., 2013; Ziegler et al., 2010).

Third, we aimed to identify potential preliterate neural markers of literacy. To approach these questions, we examined the brain–behavior relationship between vision and language and future reading fluency. Based on previous results (Dehaene‐Lambertz et al., 2018; Monzalvo et al., 2012), we restricted the analysis to faces, written, and spoken words. In general, we expected broader brain–behavior linkage for the baseline contrasts and possibly more focal activation when looking at the differential contrasts (target stimulus > all other visual conditions). More specifically, we expected that face selective responses in the left fusiform gyrus would be associated with cognitive–linguistic prereading skills and predict future reading fluency (Centanni et al., 2018, 2019). Following the results of Chyl et al. (2018), we expected that neural responses to written and auditory words in the left IOG, vOT, bilateral fusiform gyri and the MTG, and the left superior parietal lobe (SPL) would not only predict future literacy (as reported by Chyl et al., 2018) but also correlate with PA and RAN. Keeping in mind that our cohort of children was truly preliterate and had very limited letter knowledge, we considered the possibility that processing of written words might not show any brain–behavior relation. However, if sensitivity to written words in reading‐related regions like the vOT could nevertheless predict future reading fluency in the present study, this would be very strong evidence for the idea of the “reading readiness” of these regions to print prior to literacy (Dehaene & Cohen, 2007; Dehaene‐Lambertz et al., 2018).

For auditory language processing, we additionally expected interindividual differences in response selectivity in the dorsal TP language system including the planum temporale (Dębska et al., 2016; Monzalvo & Dehaene‐Lambertz, 2013). Since the bilateral STS and STG have been identified as key regions for letter speech sound integration (e.g., Karipidis et al., 2017, 2018; Richlan, 2019), we were interested in finding out whether these regions would already show response selectivity for both visual and auditory stimuli in preliterate children.

## 2 MATERIALS AND METHODS

### 2.1 Participants

In total, 90 children who were native German speakers participated in the longitudinal study investigating reading acquisition and dyslexia. All participants passed a hearing and visual acuity screening and had no history of neurological diseases. Children were recruited through advertisements in kindergartens, newsletters, Facebook groups etc. throughout the city of Berlin. Recruitment targeted children in their last year of kindergarten. Parents and children were informed that the goal was to test reading and spelling development from the end of kindergarten until the end of the second year of primary school. Children were recruited on a voluntary basis and both parents and children were carefully briefed about the longitudinal study design and the constraints of the fMRI measurement. Parents and children gave written and oral informed consent. Parents filled out a questionnaire to document their professional education level, and received travel compensation for their participation. Children were rewarded with age‐appropriate educational gifts. The Ethics Committee of the German Association for Psychology (DGPs) approved the experimental procedures (AM042014).

In Germany, formal reading instruction starts in elementary school. To ensure that children were truly preliterate, they performed a short custom‐made screening test that assessed basic letter knowledge (e.g., *a*, *d*), picture‐word matching of highly frequent words (e.g., *ball*, *cow*), syllable reading (e.g., *pa*, *som*), decoding of phonotactically valid pseudowords (e.g., *Muma*, *Ticht*) and word reading (e.g., *father*, *evening*). Three children were excluded from the study as they were able to reliably decode syllables (see Table 1 for the screening results). Furthermore, one child was excluded due to a serious specific language disorder. An additional nine children refused to participate in the training session (mock‐scanner) to familiarize children with the fMRI apparatus, resulting in 77 children participating in the passive fMRI experiment at kindergarten age (T1). Data from one child could not be analyzed due to technical problems during the scanning session and 22 children had to be excluded due to excessive movement artifacts. Consequently, the fMRI sample consisted of 54 children, 16 of these children were at family risk of developing dyslexia; that is, at least one relative was affected by developmental dyslexia as reported in a parental questionnaire (Moll & Landerl, 2010). The remaining 38 children had neither first‐ nor second‐degree relatives with developmental dyslexia. In a second session at T1, children were also tested for cognitive–linguistic prereading skills.

**TABLE 1:** Psychometric information of children before and after reading acquisition

Preliterate kindergarten children (*N* = 54)	
Age *M*(*SD*)	5.6 (.*47*)
Age range (year; month)	5;0–6;1
Female/male	28/26
Monolingual/bilingual	46/8
Maternal education^a^ *M*(*SD*)	4.4 (*2*)
Family history of dyslexia	16

	*N* items	*M* (*SD*)
*Precursors of literacy (BISC)*		
Pseudoword repetition	10	6.5 (*2.0*)
Visual attention span quality	12	11.3 (*1.1*)
Visual attention span time	10	5.4 (*3.4*)
RAN—objects^b^	8	6.2 (*1.7*)
RAN—colors^c^	12	8.4 (*3.2*)
Rhyming	10	9.2 (*1.0*)
Sound synthesis	10	9.6 (*0.8*)
Syllable segmentation	10	8.2 (*2.1*)
Sound word association	10	9.1 (*1.5*)
BISC—risk score for dyslexia	10	1.2 (*1.3*)
*Literacy screening*		
Single letter knowledge	19	8.3 (*7.2*)
Picture‐word matching	9	.60 (*1.5*)
Syllable decoding	8	.34 (*1.2*)
Pseudoword reading	8	.15 (*.63*)
Word reading	8	.09 (*.56*)

	Range	*M* (*SD*)
*Nonverbal intelligence*		
CPM ^(score)^	79^d^–146	107.3 (*18*)

Literate school children (*N* = 48)	
Age *M*(*SD*)	7.6 (.*48*)
Age range (year; month)	7;4–8;9
Female/male	27/21
Monolingual/bilingual	42/6
Handedness left/right	3/39
Maternal education *M*(*SD*)	4.4 (*2*)
Family history of dyslexia	15
Dyslexia at the end of second grade^e^	10

	Range	*M (SD)*	*r*_*s*_
*Reading and spelling skills*			
SLRT—word reading^(PR)^	1–99	49.4 (*32*)	−.45
SLRT—pseudoword reading^(PR)^	1–99	45.9 (*30*)	−.42
SLS—sentence judgment^(score)^	59–143	103.2 (*20*)	−.38
ELFE—reading comprehension^(PR)^	2–99	53.6 (*33*)	−.42
DERET—spelling ^(PR)^	0–98	42.8 (*31*)	−.44
K‐TRT—print exposure	.50–.60	.32 (.13)	
*Nonverbal intelligence*			
WISC ^(Score)^	90–147	114.5 (*13*)	

*Note*: Demographic information and behavioral test results for the fMRI samples. Means (*M*) and standard deviations (*SD*) are reported for raw data, percentile range scores (PR) or age‐normed intelligence scores (score). Min‐max values are indicated for scale of age‐normed BISC and subscales of BISC. Risk score is the aggregated information of all subscales. Spearmans rho (*r*_*s*_) are reported for the correlation of literacy tests with the BISC risk score.

^a^ Parental education was operationalized with an ordinal scale [1, without professional education [Hauptschule]; 2, Professional School, Vocational School [Realschule]; 3, High School [Gymnasium]; 4, Master Craftsman, Technical College [Fachhochschule]; 5, University Bachelor's Degree; 6, University Master's Degree; State Examination; 7, other].

^b^ Naming time is measured.

^c^ Time difference of object and color naming.

^d^ One child had a non‐verbal intelligence score < 80 at kindergarten age but reached a score of 104 at the end of the second grade.

^e^ Dyslexia was defined according to Kuhl et al. (2020): PR < 16 in at least one literacy test battery (SLRT‐II, SLS, ELFE, DERET).

To assess literacy skills, all children were invited back at the end of the second year of primary school (T2). At this point, three children from the original sample of 77 children withdrew their participation. Two children had to repeat the second grade and were retested 1 year later. Forty‐eight of these children had successfully completed the fMRI session at kindergarten age (T1). For these children, we were able to link the neural processing before onset of literacy to actual reading performance. All following results are based on the fMRI sample of 54 (concurrent analysis of cognitive–linguistic prereading skills) and 48 (longitudinal prediction of reading) children. A summary of the demographic and psychometric data is given in Table 1.

### 2.2 Psychometric measures

Psychometric assessment was conducted at two distinct developmental time points: before literacy acquisition at the end of the last year of kindergarten (T1) and at the end of the second grade of primary school (T2).

At preliterate age (T1), children were tested for non‐verbal intelligence and cognitive–linguistic skills associated with literacy. Non‐verbal intelligence was tested with *Raven's Colored Progressive Matrices* (CPM; Raven, Court, & Raven, 1998). PA, RAN, phonological short‐term memory, and visual attention control were assessed using the standardized screening *Bielefelder Screening zur Früherkennung von Lese‐Rechtschreibschwierigkeiten* (BISC; Jansen, 2002). An age‐normed risk score for the development of dyslexia was calculated for each child based on all subtests of the BISC.

At T2 (i.e., after two years of formal reading instruction), children completed a battery of standardized reading and spelling tests along with further psychometric assessments. Non‐verbal intelligence was retested with the *Wechsler Intelligence Scale for Children* (WISC‐IV; Petermann & Petermann, 2014). We added measures of verbal development (scope of lexicon) and non‐verbal working memory (digit span) also using the WISC‐IV. To characterize literacy development, speed and accuracy of single word and pseudoword reading were tested (two subtests of the *Lese‐ und Rechtschreibtest II—Weiterentwicklung des Salzbuger Lese‐ und Rechtschreibtest*; SLRT‐II; Moll & Landerl, 2010). Reading speed and comprehension on the sentence level were evaluated using a plausibility judgment test (Salzburger Lese‐Screening für die Klassenstufen 1–4; SLS; Mayringer & Wimmer, 2008). The age‐normed SLS score is a combined score capturing reading speed and accuracy with a scaling that is equal to the intelligence quotient. Furthermore, reading comprehension was tested on three levels of increasing complexity, from word meaning to text comprehension (ELFE 1–6: *Ein Lesesinnverständnistest für Erst‐ bis Sechstklässler*; Lenhard & Schneider, 2006). Finally, writing and spelling skills were assessed using a short spelling task (DERET 1–2+: Deutscher Rechschreibtest für das erste und zweite Schuljahr; Stock & Schneider, 2008). Psychometric assessment was completed by testing PA (Basiskompetenzen für Lese‐Rechtschreibleistungen: BAKO 1–4; Ein Test zur Erfassung der Phonologischen Bewusstheit vom ersten bis vierten Grundschuljahr; Stock, Marx, Schneider, & Schneider, 2003; subtests: phoneme transition, sound categorization, vocal length) and children's exposure to books (*K‐TRT: Kinder Titelrekognitionstest*; Schroeder, Segbers, & Schröter, 2016). Finally, handedness was assessed as the hand with which children wrote.

### 2.3 fMRI stimuli

During the passive fMRI paradigm, five different sensory stimuli were presented, targeting visual symbol and spoken language processing. Auditory stimuli consisted of spoken words (*N* = 60) generated with MAC OSX voice Anna (female). Visual stimuli encompassed horizontal checkerboards (*N* = 15), houses (*N* = 60), faces (*N* = 60), and written words (*N* = 60). To match the visual angle across conditions, all stimuli were presented in gray scale and uniform size in the middle of the screen. Faces (male and female) were taken from the *Karolinska Directed Emotional Faces* (KDEFF; Lundqvist, Flykt, & Öhman, 1998) and *Radboud Face Database* (RaFD; Langer et al., 2010) databases. All faces had a neutral expression, forward gaze and were Caucasian. Visually and auditory presented words were highly frequent (absolute type frequency: spoken words range = 61–1971, *M* = 233, *SD* = 305; written words range: 60–1,355, *M* = 180, *SD* = 179) mono‐syllabic nouns of four to six letters and were taken from the childLex database, which provides age‐specific norms for children (www.childlex.de; Schroeder, Würzner, Heister, Geyken, & Kliegl, 2015; corpus range of absolute type frequency: 1–9, 255). Here, we chose the youngest reference group of six‐ to eight‐year‐old children (beginning readers, grades 1–2). Spoken and written words were carefully matched for lemma, bigram and neighborhood frequency.

### 2.4 fMRI design and data acquisition at kindergarten age

To familiarize children with the apparatus, sounds and requirements of fMRI testing, children performed a training session prior to the actual fMRI experiment in a mock‐scanner at the *Max Planck Institute for Human Development* (see Raschle et al., 2012, for an outline). During the training, we explained the functioning of the scanner, the experimental pipeline, and the negative effects of extensive head movement on scanning results. Approximately a week later, children returned for a second session at the *Center for Cognitive Neuroscience Berlin* (CCNB), in which the actual fMRI experiment was conducted. The experiment was divided into four runs, with the four visual and the auditory conditions presented block‐wise (15 stimuli per block). To ensure children's attention, a catch trial was performed at the beginning of each block (150 ms), in which a picture of the mascots of the project was shown and children were instructed to press a button using their middle or index finger to confirm its appearance. Average performance on the catch trials for the cohort of 54 kindergarten children was .67 (range = 0–1, *SD* = .31). As indicated by the range of performance, the mean value was strongly influenced by a few children that did not manage to respond; they may possibly have been mentally overstrained by the highly new and demanding situation, or simply forgot to press the button. In any case, when asked afterwards whether they noticed the mascot, all of these children confirmed that they had. As such, the data from those children was kept for further analyses. Catch trials were followed by a jittered fixation cross (300–600 ms) and then replaced by the first stimulus. Visual stimuli were shown for 500 ms and auditory stimuli were presented for 500–600 ms. Each stimulus was followed by an inter‐stimulus interval of 200 ms (fixation cross). Three to five null‐events were included in each run to allow the blood‐oxygenation‐level dependent (BOLD) signal to return to baseline (2–14 s, 26 s per run). Each run took about 136 s and was followed by a short break to improve compliance and maintain attention of the children (see Figure S1 for a schematic overview of the fMRI design). The order of the stimuli and null‐events was optimized using the optseq2 algorithm (Dale, 1999). The order of blocks was pseudorandomized within runs, and the order of runs was counterbalanced across subjects. The session was completed by a high‐resolution structural scan during which children could relax and watch a short movie. The entire scanning session lasted for about 15 min.

Imaging was performed using a 3.0 T Siemens Magnetom Tim Trio scanner (Siemens, Erlangen, Germany) equipped with a 12‐channel head coil. In each of the four runs, 68 whole brain functional T2*weighted echoplanar (EPI) pulse sequences (TE: 30 ms, TR: 2000 ms, 70° Flip Angle, 37 slices, matrix: 64 × 64, field of view: 192 mm; 3 × 3 × 3 mm^3^ voxel size, 20% interslice gap) were acquired, resulting in a total of 272 axial volumes. Additionally, a T1‐weighted matched‐bandwidth high‐resolution anatomical scan with the same slice prescription as EPI was acquired (176 sagittal sections, 2 × 2 × 2 mm^3^ voxel size, matrix: 256 × 256). The standardized pictures were presented at the center of a white screen on dual display goggles (VisuaStim, MR Research) using Python 2.7 (Python Software Foundation). Auditory stimuli were presented via circumaural earphones (VisuaStim, MR Research). To attenuate scanner noise, child‐appropriate earplugs were provided, and the children's heads were padded with foam to improve their comfort and reduce motion artifacts.

### 2.5 fMRI data analyses

FMRI data was preprocessed and analyzed using the SPM12 software package (Wellcome Department of Imaging Neuroscience, University College London, UK, 2014). Images were spatially realigned to the first volume by means of rigid body transformation, and unwarped. A confound of testing very young children with an fMRI paradigm is that head motion is highly correlated with age and further developmental characteristics (Satterthwaite et al., 2012), leading to increased motion artifacts. Consequently, the Artrepair toolbox (Mazaika, Hoeft, Glover, & Reiss, 2009) was used to detect outlier volumes in which scan‐to‐scan motion was greater than 1.5 mm (Karipidis et al., 2017; Pleisch et al., 2019). Outlier volumes were replaced by interpolating with the preceding and following correct images. Children with more than 10% repaired images (Pleisch et al., 2019) were excluded from further analysis (*N* = 22). Overall, less than 2.8% of the data was repaired. Tissue probability maps for native space components of the structural images were created according to an age‐matched pediatric template using the Template‐o‐Matic toolbox (Wilke, Holland, Altaye, & Gaser, 2008). The nonlinear Fast Diffeomorphic Anatomical Image Registration Algorithm (DARTEL; Ashburner, 2007) was used to create a study‐specific template. Subsequently, transformation from this study‐group specific template to MNI space was estimated. Finally, functional images (voxel size 2 ***×*** 2 ***×*** 2 mm^3^) were spatially smoothed with an 8 mm (FWHM) Gaussian kernel.

In a next step, individual fixed‐effect models were computed using the default value of the high‐pass filter (128 s) which partitions out the confounding influence of physiological noise. Experimental conditions were entered into a general linear model (GLM) and motion parameters generated during realignment were included as regressors of no interest to control for overall motion effects. Basic contrast maps (target stimulus against null) were generated for each stimulus condition (baseline conditions, hereafter denoted as: *checkerboards*, *houses*, *faces*, *written words*, and *spoken words*). First, we examined the baseline contrast of each stimulus condition by entering the single‐subject maps into second level one‐sample t‐tests using the flexible factorial model of SPM. Similar to the results of Dehaene‐Lambertz et al. (2018), *checkerboards* elicited larger activation than the other categories (bilateral calcarine: *k* = 12,784, *T* = 23.8, *p*_*FWE*_ < .001, [−8–90 4], right SPL: *k* = 351, *p*_*FWE*_ < .03, *T* = 7.96, [24– 70 44]). This was most certainly due to perceived movement induced by the fast‐changing line orientations from trial to trial and *checkerboards* were discarded from further analysis. Consequently, the specific neural response to *houses*, *faces* and *written words* was extracted by subtracting the activation of the other two remaining visual conditions (*houses* > [*faces*, *written words*], *faces* > [*written words*, *houses*], *written words* > [*faces*, *houses*]; cf. Dehaene‐Lambertz et al., 2018; Monzalvo et al., 2012 for a similar approach). The differential contrast of *written words* > [*faces*, *houses*] did not yield any significant activation and was thus not included in the whole brain–behavior association analysis. In a second step, single‐subject contrast maps of the baseline contrasts (*faces*, *written words*, *spoken words*) and the differential contrast *faces* > [*written words*, *houses*] were entered into group level regression analyses. Here, results of the behavioral tests were entered as regressors to evaluate (A) the relationship between cognitive–linguistic prereading skills (PA, RAN) and basic visual and auditory processing and (B) the power of preliterate neural processing to predict reading fluency after two years of formal reading instruction. Significant neural activation was inspected on the whole brain level with an initial cluster‐defining threshold of *p <* .001 (uncorrected) and a second family wise error (FWE‐)corrected cluster‐level extent threshold, measured in units of contiguous voxels (*k*), of *p*_*FWE*_ < .05 corrected for multiple comparisons across the set of analyzed voxels (Flandin & Friston, 2019; Mueller, Lepsien, Möller, & Lohmann, 2017; Woo, Krishnan, & Wager, 2014). To avoid false positives, regression analyses were additionally controlled for the number of tests performed resulting in *p* < .006 for the concurrent correlational analysis between neural activity, PA and RAN (4 fMRI contrasts × 2 cognitive–linguistic skills) and *p* < .0125 for the longitudinal prediction of reading (4 fMRI contrasts × 1 reading skill) denoted as *p*_*corr*_. For the sake of comparability with previous studies (e.g., Chyl et al., 2018; Dehaene‐Lambertz et al., 2018; Monzalvo et al., 2012; Pollack & Price, 2019), we report the results for both: the stricter *P*‐values additionally accounting for multiple testing and also the standard correction accounting for the number of voxels (FWE). Brain regions are reported according to the Montreal Neurological Institute (MNI) space brain atlas.

As stated above, the differential contrast *written words* > [*faces*, *houses*] did not yield any significant results on the whole brain level. Thus, we decided to run a more focal region of interest (ROI) analysis to capture more subtle effects of the brain–behavior relationship. Literature‐based ROI analysis was computed for the bilateral fusiform, MTG, and the STG; the left IOG, and the SPL. All ROIs were anatomically defined using the *aal* atlas of the *wfupickatlas* (Maldjian, Laurienti, & Burdette, 2004; Maldjian, Laurienti, Burdette, & Kraft, 2003; Tzourio‐Mazoyer et al., 2002). The significance threshold for the ROI analysis was set to *p <* .001 (uncorrected) and *k* > 10 voxels. To account for the number of regression models, we report the results at a stricter threshold of *p*_*corr*_ *<* .0005 for the concurrent brain–behavior analysis (1 fMRI contrast × 2 cognitive–linguistic skills). For the longitudinal prediction of reading fluency, no further correction was required (1 fMRI contrast × 1 reading skill). A figure of the ROIs and the results of the ROI analyses for all other contrasts is provided in the supplementary materials (Figure S2; Tables S3 and S4).

### 2.6 Behavioral data analyses

Behavioral data was analyzed using R Studio (Version 1.1.453; R Core Team, 2020; R version 3.5.0) complemented by *afex* (Singmann & Klauer, 2020), *car* (Fox & Weisberg, 2019), *corpora* (Evert, 2015), *Hmisc* (Harrell, 2019), *lmtest* (Zeileis and Hothorn, 2002), and *psych* (Revelle, 2020). For statistical analyses, psychometric data and demographic information was standardized or centered when appropriate. Spearman rank correlations of the age‐normed combined score of preliterate measures (BISC risk score for dyslexia; Jansen, 2002) collected at T1, and the age‐normed reading and spelling tests (T2) were computed. To account for multiple testing, Holm‐corrected *P*‐values are reported.

For the fMRI regression analyses, results of the two RAN subtests (objects, colors; BISC, Jansen, 2002) were combined by taking the mean. Likewise, one metric was formed as an indicator of reading fluency (cf. Karipidis et al., 2018) consisting of word and pseudoword reading of the SLRT‐II (Moll & Landerl, 2010). Furthermore, studentized residuals of *z*‐standardized PA, RAN and reading fluency were computed to control for confounding effects of sex and non‐verbal intelligence before being entered into the regression.

## 3 RESULTS

### 3.1 Behavioral predictors of future literacy

The correlation between preliterate risk for dyslexia at T1 and reading and spelling measures at T2 showed that weak performance on the various preliteracy component measures (indicated by a higher risk score of the aggregated BISC; Jansen, 2002) was significantly associated with lower literacy proficiency 2 years later (see Table 1). A detailed correlation matrix is provided in the supplementary material (Figure S3).

### 3.2 Neural system of vision and spoken words in kindergarten

**FIGURE 1:**
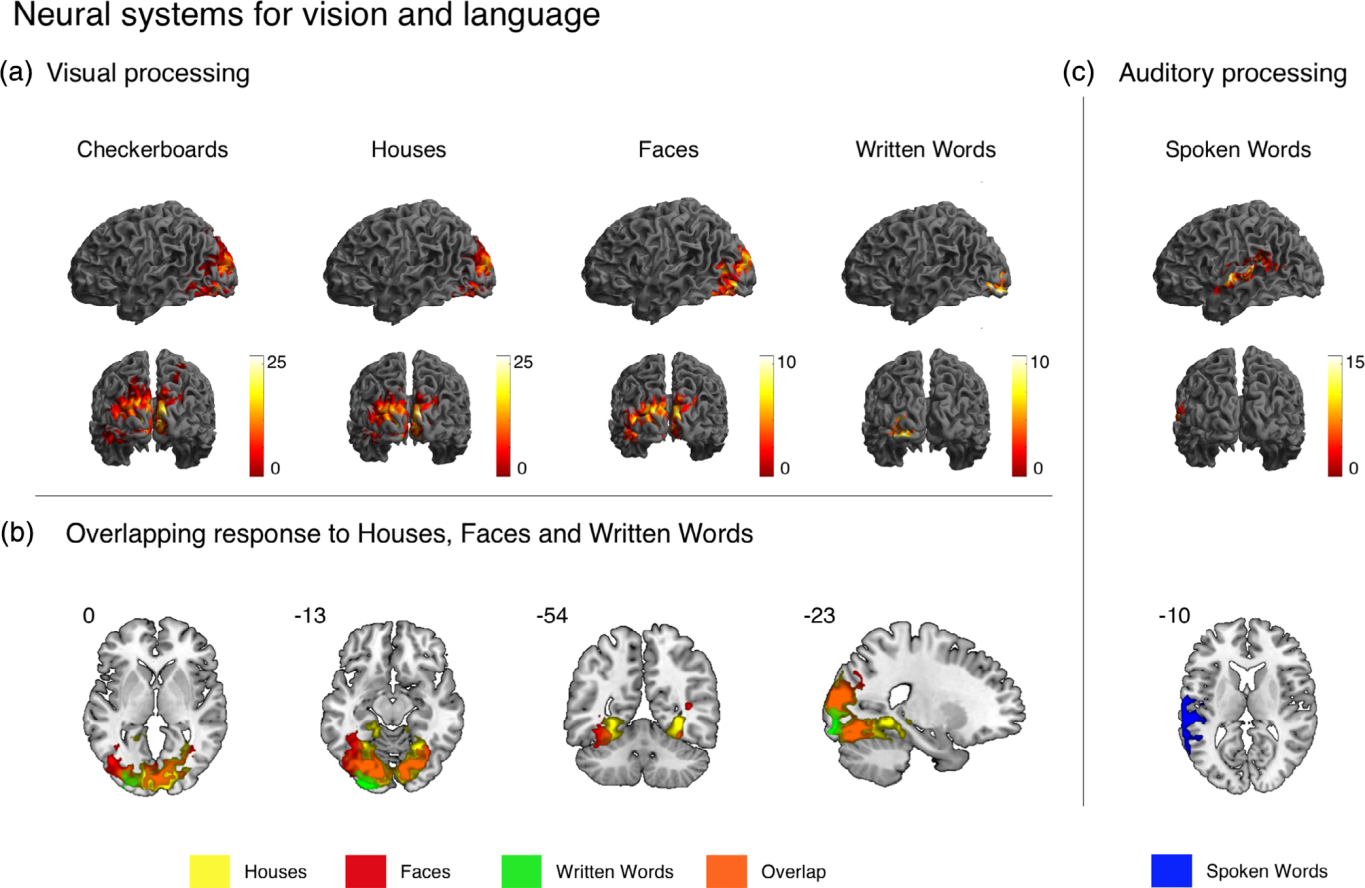
Baseline contrast maps of visual and auditory stimuli for 54 preliterate children. (a) Whole brain rendering for all visual stimulus conditions. Colorbars indicate *T*‐values for each contrast. (b) Selected slices showing overlap of neural activity in response to baseline contrasts of *houses* (yellow), *faces* (red) and *written words* (green); overlay is depicted in orange. (c) Whole brain rendering and exemplary axial slice (blue) for neural response to *spoken words*. Significant activation is reported at *p* < .001 (uncorrected) and FWE‐corrected on cluster level at *p* < .05

Figure 1 shows the neural activation of all baseline contrasts (stimulus condition > 0). FMRI results are presented on *p* < .001 (uncorrected) and *p*_*FWE*_ < .05. As predicted, *houses* and *faces* elicited a neural response in the ventral visual stream. *Houses* showed activation in the bilateral IOG reaching to the left occipital fusiform gyrus (OFuG; *p*_*FWE*_ < .001, *k = 9,744*, *T* = 19.7, [12 –94 12]). *Faces* elicited left‐lateralized activation in the IOG (*p*_*FWE*_ < .001, *k = 7,639*, *T* = 12.6, [−14 –96 16]). *Written words* showed a small cluster in the left OFuG to IOG (*p*_*FWE*_ < .007, *k = 518*, *T* = 8.51, [−26 –92 −14]). A large bilateral cluster in the occipital cortex was activated for *houses* > [*faces*, *written words*] (*p*_*FWE*_ < .001, *k* = 10,340, *T =* 21.4, [12 –92 12]). The *faces* > [*houses*, *written words*] contrast was associated with significant bilateral activation in the cuneus reaching to left lingual gyrus (*p*_*FWE*_ < .001, *k* = *1871*, *T* = 9.95, [0 –82 22]) and the supplementary motor cortex (*p*_*FWE*_ < .002, *k* = *670*, *T* = 6.44, [0 –8 48]). The contrast *written words* > [*houses*, *faces*] did not yield any significant response.

As expected, the baseline contrast of *spoken words* activated the dorsal language stream associated with significant activation in the left STG (*p*_*FWE*_ < .001, *k* = *1921*, *T* = 14.3, [−60 –10 0]). Detailed tables of all baseline and differential contrasts are provided in the supplementary materials (Tables S1 and S2).

### 3.3 Concurrent brain–behavior relationship

Several regression models were computed to evaluate whether neural activation in response to visual symbols and spoken language was related to interindividual differences in PA and RAN (cf. Section 2.2 for a detailed description of all subtests) evaluated at the same timepoint (cf. Table 2 for cluster size, peak activation and location of the brain–behavior relationship). Here, the results are presented at two different significance levels (see Section 2.5 *fMRI data analyses* for a detailed description): *p*_*corr*_ < .006 controlled for the number of regression models, and the standard threshold of *p*_*FWE*_ < .05.

**TABLE 2:** Association of neural processing and rapid automatized naming

	Anatomical location	MNI			Cluster		Peak
		*x*	*y*	*z*	*p*_*FWE*_	*k*	*T*
Visual processing							
Positive correlation with **Faces > [Houses, Words]**							
Ventral	L fusiform	−42	−44	−18	.005**	227	4.51
	*L occipital fusiform*	−36	−66	−14			3.98
	R fusiform	30	−58	−12	<.001**	428	5.79
	*R lingual gyrus*	12	−86	−18			5.63
	*R occipital fusiform*	34	−76	−6			5.24
	L middle frontal	−28	8	46	.008*	204	4.79
Negative correlation with **Written Words**							
Dorsal	L superior temporal gyrus	−56	−52	20	.002**	309	4.76
	*L supramarginal*	−44	−42	38			4.68
	R angular gyrus	50	−46	30	.050*	145	5.04
	*R supramarginal*	44	−42	22			4.17
	R caudate	26	6	18	.033*	163	4.52
	*R middle frontal*	32	10	36			3.85
Auditory processing							
Negative correlation with **Spoken Words**							
Dorsal	L precuneus	−8	−56	46	<.001**	456	5.70
	*L superior parietal*	−16	−62	54			4.26
	*R precuneus*	4	−54	54			3.72
	L precentral	−56	4	14	.001**	339	4.96
	*L postcentral*	−46	−18	28			4.26
	*L central operculum*	−46	−16	16			4.20

*Note*: Results of independent fMRI analyses for baseline and differential contrasts on group‐level with rapid automatized naming as regressor of interest. Clusters are ordered according to language‐reading system. MNI = coordinates of cluster center of mass, *k* = number of voxels; *T* = *T*‐value of peak activation.

* Significant at *p*_*FWE*_ < .05.

** Significant at *p*_*corr*_ < .006 additionally corrected for multiple testing.

**TABLE 3:** Prediction of literacy

	Anatomical location	MNI			Cluster		Peak	ES
		*x*	*y*	*z*	*P*_*FWE*_	*k*	*T*	*d*
Visual processing								
Positive relationship with **Faces**								
Ventral	L inferior occipital	−40	−66	4	.008**	193	4.21	.61
	*L middle temporal*	−52	−68	4			4.09	.59
Dorsal	L Precuneus	−12	−48	44	.028*	146	4.09	.59
	*L angular gyrus*	−32	−66	48			3.90	.56
	*L superior parietal*	−18	−62	44			3.89	.56

*Note*: Results of independent fMRI analyses for baseline and differential contrasts on group‐level with reading fluency as regressor of interest. Clusters are ordered according to language‐reading system. MNI = coordinates of cluster center of mass, *k* = number of voxels, *T* = *T*‐value of peak activation, ES = effect size, *d =* Cohen's *d* (.5 = medium effect size).

* Significant at *p*_*FWE*_ *<* .05.

** Significant at *p*_*corr*_ < .0125 corrected for multiple testing.

**FIGURE 2:**
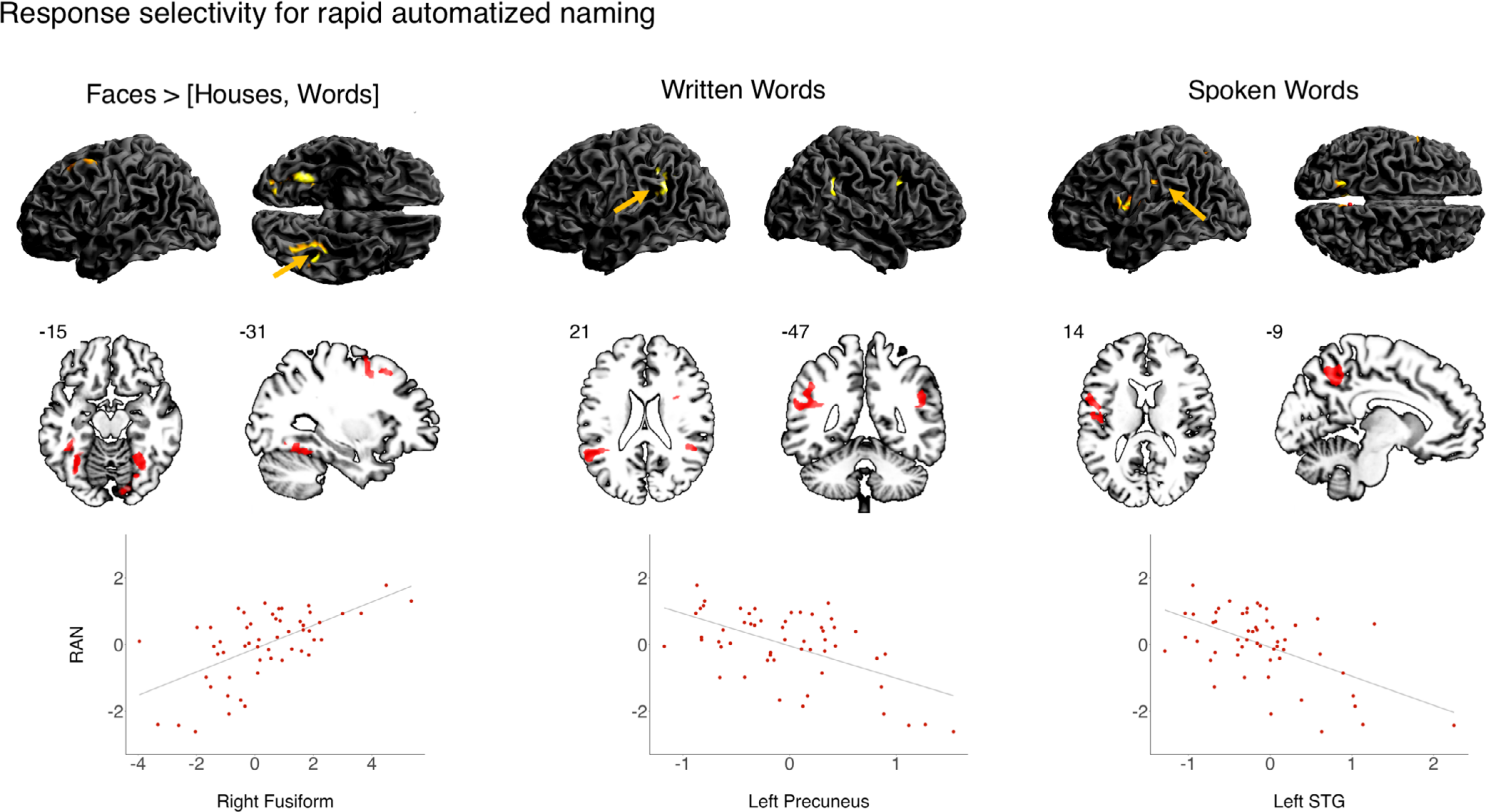
Association of neural response to *faces* > [*houses*, *words*] and baseline contrasts of *written words* and *spoken words* and rapid automatized naming (RAN) in 54 preliterate children. Significant activation is reported at *p* < .001 (uncorrected), FWE‐corrected on cluster level at *p* < .05, additionally controlled for number of regression models at *p* < .006. Whole brain rendering and exemplary scatter plots of the neural response are shown for right fusiform gyrus, left superior temporal gyrus (STG) and left precuneus (studentized residuals of beta values in arbitrary units averaged across region of interest; *x*‐axis and *z*‐standardized studentized residuals of RAN; *y*‐axis). Yellow arrows indicate the plotted regions

As shown in Figure 2, the association analyses of the neural response to *faces* > [*houses*, *written words*] in relation to RAN performance showed a positive correlation, meaning that higher neural activity was correlated with higher RAN speed. This association was found in the right fusiform gyrus extending into the IOG at *p*_*corr*_. At *p*_*FWE*_ two further clusters were identified: the left fusiform gyrus and the left middle to superior frontal gyrus. The baseline contrast of *faces* did not yield a significant association with RAN. *Written words* was negatively correlated with RAN in the left SMG and the AG at *p*_*corr*_ and the right caudate as well as the right AG to SMG at *p*_*FWE*_. *Spoken words* was negatively associated with RAN in the left precuneus extending to SPL at *p*_*corr*_ and the left precentral at *p*_*FWE*_. PA was not systematically associated with any stimulus condition.

### 3.4 Longitudinal neural predictors

**FIGURE 3:**
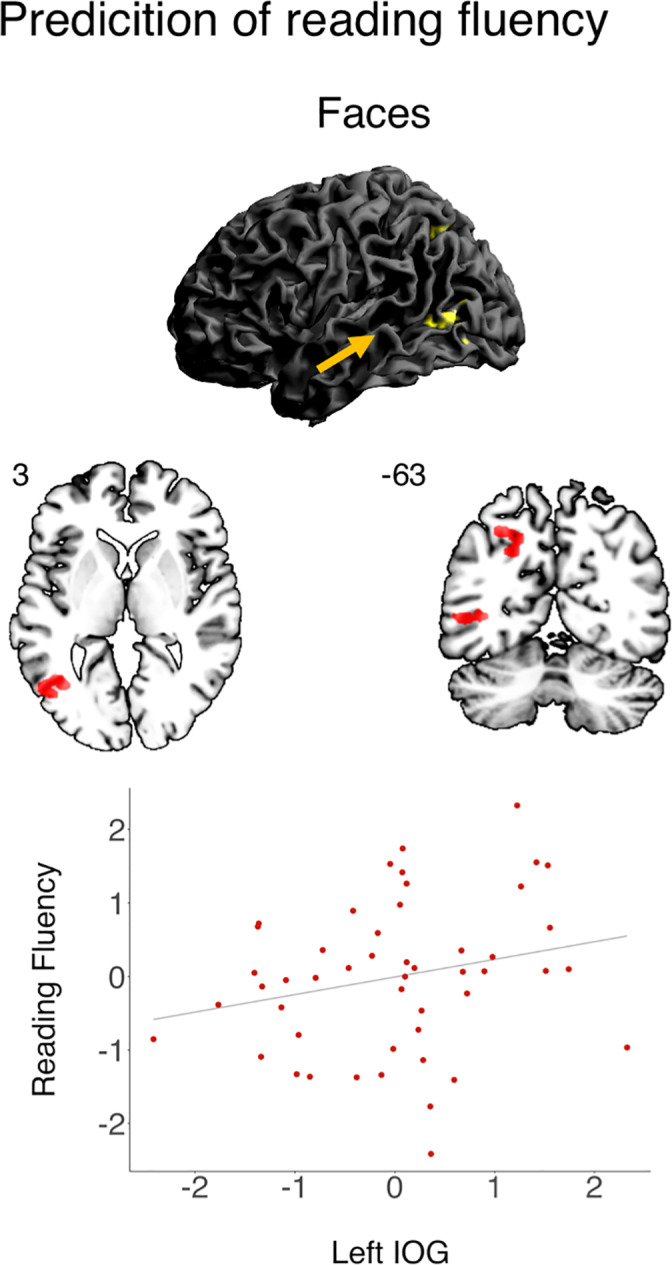
Association of neural response to baseline contrast of *faces* and reading fluency in 48 school children. Significant activation is reported at *p* < .001 (uncorrected) and FWE‐corrected on cluster level at *p* < .05, additionally controlled for number of regression models at *p* < .0125. Whole brain rendering and an exemplary scatter plot of neural response in left occipital temporal gyrus (IOG; studentized residuals of beta values in arbitrary units averaged across region of interest; *x*‐axis) and *z*‐standardized studentized residuals of reading fluency (*y*‐axis) is shown

Next, we explored the predictive power of neural processing of visual symbols and spoken language before literacy acquisition on future reading fluency at *p*_*corr*_ < .125 and *p*_*FWE*_ < .05. Visual processing of *faces* (baseline contrast) at kindergarten age explained interindividual variance in word and pseudoword reading (see Figure 3 and Table 3). The higher the neural activation in response to faces in kindergarten, the better the reading performance two years later. Specifically, the BOLD response to *faces* in left IOG, ITG and MTG was associated with reading fluency (at *p*_*corr*_). At *p*_*FWE*_ the left precuneus, AG and SPL also yielded a significant association. None of the other contrasts tested predicted reading fluency (baseline contrasts of *written words and spoken words*, differential contrast of *faces* > [*houses*, *words*]).

### 3.5 ROI analyses for print‐specific activation

Using more focal ROIs to examine the brain–behavior relationship for the differential contrast *written words* > [*faces*, *houses*], we found a negative correlation for a small cluster in the left STG (*p*_*corr*_ < .0005, *k* = 17, *T =* 3.98, [−56 –46 18]) and RAN. No further ROI yielded a significant brain–behavior relationship with RAN, PA or future reading fluency.

## 4 DISCUSSION

Literacy is a fundamental skill required in everyday life, academic education, and across the span of a career (Kendeou, McMaster, & Christ, 2016). However, many children struggle to solve even relatively easy reading tasks (Artelt, Schiefele, Schneider, & Stanat, 2002; Elleman & Oslund, 2019). With our longitudinal approach, we examined possible neural markers that might help to identify children who may be at risk of encountering reading difficulties later on. Our key findings can be summarized as follows: Brain activity elicited by our passive viewing and listening tasks was sensitive to interindividual differences in preliterate cognitive–linguistic skills and predicted future reading. More specifically, RAN performance was positively correlated with face encoding in the ventral stream and negatively associated with auditory language processing in the dorsal stream. Interestingly, when examining the neural response to written words in preliterate children with very limited letter knowledge, we also found response selectivity to RAN in the left STG (negative association), a region that tends to be strongly involved in audio‐visual integration (Karipidis et al., 2017, 2018; Richlan, 2019). Most importantly, neural response to faces in the ventral stream clearly predicted future reading fluency two years later. In sum, we observed fine interindividual differences in the neurofunctional architecture of the not‐yet‐established ventral and dorsal reading streams in relation to cognitive–linguistic skills associated with literacy and future reading in preliterate children. These differences in neural “reading readiness” might reflect different starting points that children are at when formal literacy instruction starts.

### 4.1 Concurrent brain–behavior relationships

The cognitive–linguistic skills associated with literacy are well described (Landerl et al., 2013, 2019; Ziegler et al., 2010). Their neural underpinnings, however, have been less extensively studied (for exceptions, see Karipidis et al., 2017; Monzalvo et al., 2012; Turkeltaub et al., 2003). In the first part of our brain–behavior analysis, we aimed to identify neural regions that are sensitive to PA and RAN known to predict future reading (Landerl et al., 2013, 2019) and might thus mark the neural “reading readiness” at the end of kindergarten. The whole brain association analyses showed a link between the neural functioning of vision and language, and interindividual differences in RAN performance. Rapid naming of objects and reading fluency share many subprocesses, such as saccadic eye movement, working memory, lexical access and mapping visual objects onto language representations (Norton & Wolf, 2012). Not surprisingly, RAN has been shown to be a strong predictor of later reading fluency across orthographies (Georgiou, Parrila, Cui, & Papadopoulos, 2013; Landerl et al., 2019). In line with our hypotheses, enhanced face‐selective bilateral fusiform activation correlated with higher RAN performance in our cohort of kindergarten children. Our results extend the findings of Monzalvo et al. (2012), who reported that face‐selective responses in the right fusiform correlated with reading fluency in children with varying reading abilities, to younger preliterate children. Furthermore, recent studies showed that print‐sensitive left‐hemispheric vOT activation is positively correlated with reading fluency in emergent readers (e.g., Brem et al., 2020; Chyl et al., 2018; Dehaene‐Lambertz et al., 2018), and also predicts future literacy in prereaders (Centanni et al., 2018; Karipidis et al., 2018). These findings are in line with an assumed dysfunction in left vOT in children with dyslexia (see Richlan, Kronbichler, & Wimmer, 2011 for a meta‐analysis), which is also accompanied by structural differences (Hoeft et al., 2007). The current findings extend those from previous studies by showing that not only neural responses to print, but also neural response to (nonlinguistic) faces in the ventral stream is associated with reading‐relevant skills in prereaders, that is, with the measure of RAN known to facilitate reading acquisition (Landerl et al., 2013, 2019).

We found that neural response to written words was associated with RAN in the left dorsal stream; however, no such association was found in the ventral stream. This finding might seem surprising, as our children were truly preliterate and were not able to actually decode the written words. Nevertheless, we saw a negative correlation in the left STG and SMG for written words in relation to RAN, which was supported by a more focal ROI analysis for print‐specific response. This finding indicates that words elicit a neural response in a region that rapidly becomes sensitive to audio‐visual integration during reading acquisition (Karipidis et al., 2017, 2018), even when children are not yet literate. Similarly to reading, sequentially naming objects in a rapid naming task involves the mapping of visual symbols onto spoken language. Thus, the observed sensitivity of the left STG and SMG might mark a child's ability to set up a network that maps visual information onto phonology.

As predicted, processing of spoken language was sensitive to RAN performance in the dorsal stream, that is, the left precuneus and SPL. Similarly to Chyl et al. (2018), who reported a negative correlation between the dorsal spoken language stream and reading skills in emergent readers, we found weaker neural responses to the two linguistic categories (written and spoken words) to correlate with higher RAN performance in preliterate children. In our opinion, there are at least two possible explanations for the observed brain–behavior relationship. As recently reported, the bilateral precuneus shows developmental decreases during phonological processing in prereaders compared to emergent readers (Yu et al., 2018). Our results thus agree with the hypothesis that less effortful spoken language processing facilitates phonologically based cognitive–linguistic preliterate skills. Another possibility is that our passive viewing and listening task simply tapped into the TP attention network (Vossel, Geng, & Fink, 2014). According to this interpretation, children who had attentional difficulties in keeping track of the stimuli might also exhibit attentional and executive problems when asked to rapidly name objects. Since guiding one's attention and executive functioning are pivotal parts of reading acquisition, both interpretations agree with our hypothesis that preliterate response‐selectivity in the dorsal stream forms a neural underpinning of successful RAN performance, which, in turn, is a powerful predictor of reading.

Besides the brain–behavior relationship with the dorsal language system, RAN was also negatively correlated with neural response to spoken words in the left precentral gyrus. Left pre‐ and postcentral gyri have been identified as essential convergence zones for visual and articulatory representations, that is, speech production (Monzalvo et al., 2012; Price, 2010). Indeed, RAN requires moving from visual encoding to retrieval of lexico‐semantic representations and articulatory sequencing, which is exactly what happens during speech production. Emergent readers in particular strongly rely on phonological recoding during their first steps of reading acquisition (Goswami, 2011; Ziegler, Bertrand, Lété, & Grainger, 2014) and RAN might at least partly test the preliterate functioning of the underlying neural circuit. Against our hypotheses, we did not see any systematic relationship between neural response selectivity and PA performance. This could either indicate that our passive viewing and listening task was simply not suitable to detect interindividual differences in PA, or may demonstrate that RAN is a stronger cognitive–linguistic predictor for transparent orthographies (Landerl et al., 2013, 2019).

In sum, we found interindividual differences in neural sensitivity to RAN, one of the most important behavioral predictors of literacy, solely in those regions that will become key components of the ventral and dorsal reading stream during literacy acquisition. In general, our analysis does not allow conclusions about the causality of the observed brain–behavior relationships to be drawn. Application of predictive modeling approaches using separate data sets for training and testing might be a promising tool to shed further light on the trajectories of preliterate functioning of future reading streams (e.g., Feng et al., 2020).

### 4.2 Longitudinal neural predictors of future literacy

The longitudinal design of our study allowed us to investigate to what extent neural processing of vision and language obtained at kindergarten age is sensitive to literacy after two years of reading instruction. Indeed, neural responses to faces in the ventral stream clearly predicted future reading fluency. In contrast, neural sensitivity to written words did not predict reading fluency in our cohort of children. This could indicate that sensitivity to written words (which are a cultural invention) have no special status as predictors of later reading ability prior to reading instruction. The initial stages of successful reading acquisition consist primarily of learning how to phonologically decode words (Share, 1995; Ziegler et al., 2020). Mere sensitivity to written words prior to reading might be too distant from the core phonological decoding processes to show a brain–behavior relationship. Recently, Chyl et al. (2018) reported weak activation in response to print in the left ventral stream and its right‐hemispheric homologues in preliterate children. However, as they did not explicitly test for the predictive power of this print‐sensitive activation in the bilateral vOT, and as we did not observe any print‐selective response sensitive to predict reading, the question of whether preliterate print‐specific response in the ventral stream can serve as a neural marker for reading development, remains open to be tackled in future research. In contrast to previous studies (e.g., Yu et al., 2018) and against our hypothesis, auditory language processing was not systematically associated with future reading fluency. Perhaps the passive listening task was not challenging or engaging enough to sufficiently trigger interindividual differences in the neural response to spoken language. However, the observed reading‐sensitive response to faces in left IOG to MTG could be clearly seen as a preliterate neural marker for future reading acquisition. Similarly, older literate children with dyslexia previously showed a reduced response to faces in the right‐hemispheric fusiform gyrus that was furthermore correlated with reading skill (Monzalvo et al., 2012). The theory that face and word recognition share the exact same cortical circuits (Behrmann & Plaut, 2013; Dehaene & Cohen, 2007) has recently been questioned (Dehaene‐Lambertz et al., 2018). Reading might instead encroach on formerly weakly specialized neurons of the fusiform gyrus lateral to the face area. The question remains of why neural response to faces in the ventral visual cortex would be a reliable predictor of successful reading acquisition. First, the vOT is strongly connected to the left‐hemispheric spoken language system (Gomez et al., 2017; Saygin et al., 2016). Second, neurons of the vOT have a high plasticity, which manifests in the fine‐tuning, even in adulthood, to behaviorally relevant stimuli (De Heering & Rossion, 2008; Mongelli et al., 2017). These two factors are in favor of the observed brain–behavior relationship of preliterate face encoding and future literacy. A decisive prerequisite for alphabetic reading acquisition is to gain insights into the phonological structure of spoken words, as letters represent phonemes (Goswami, 2000; Ziegler & Goswami, 2005). Consequently, facial speech movements might be of particular interest both shortly before, and during, reading acquisition in order to discover and represent phonemes (see Sekiyama & Burnham, 2008, for an increased McGurk effect in emergent readers). Face encoding is already well established and reproducible by the age of five (Dehaene‐Lambertz et al., 2018). The observed positive correlation between face processing in the fusiform gyrus and RAN and the association between face sensitive response in the vOT and future reading fluency might therefore reflect an increased attention to articulation, which supports reading acquisition. One possibility is that the observed selective response to RAN and reading fluency in bilateral fusiform and the left MTG during face processing is due to the functional neuroanatomical prerequisites of the ventral stream, that is, high neural plasticity and pre‐existing connectivity to the language system. Another possible explanation might be that it mirrors attentional processes facilitating the discovery of the relevant cognitive–linguistic skills associated with literacy, that is, insight into the phonological system of language. Either way, preliterate neural response to faces in the ventral stream might be a promising objective neural marker of future reading performance.

## 5 LIMITATIONS AND OUTLOOK

Due to the very young age of the children, we kept the fMRI tasks as short as possible at the expense of data points per subject. Similarly, we kept the number of stimulus conditions to a minimum to increase feasibility, and thus could not include control conditions for each stimulus. Additionally, we had to exclude checkerboards as a baseline condition from analysis due to atypically large activations. However, we believe that the relatively large number of participants and the rapid block design ensured stable and valid results. Likewise, motion artifacts are inevitable when scanning pediatric cohorts. Although we carefully checked the data and applied rather strict motion correction, a possible confound cannot be entirely ruled out. Although differences in brain sizes between children and adults are negligible by the age of six (Kang, Burgund, Lugar, Petersen, & Schlaggar, 2003), mapping a child's brain onto MNI space bears the risk of introducing confounds. This remains a methodological issue that needs to be tackled. With a mean intelligence score of 115 our cohort of children represents the upper limit of the population. To account for this bias, all statistical analyses were controlled for non‐verbal intelligence. From a cross–linguistic perspective, it would be interesting to see a replication of our results in deeper orthographies or nonalphabetic writing systems.

## 6 CONCLUSION

In summary, we have shown that the neural systems of vision and spoken language are associated with interindividual differences in cognitive–linguistic preliterate skills. In prereaders, RAN performance was correlated with face‐specific responses in the bilateral fusiform gyrus, and with written and spoken language processing in the dorsal stream. Our results emphasize an early sensitivity to cognitive–linguistic preliterate skills in regions that later become integral parts of the developing and proficient reading network, in particular audio‐visual convergence zones like the STG. Returning to the initial research question of whether specific preliterate neural correlates are sensitive to future literacy, we conclude that face processing showed a selective response in the left vOT which predicted reading fluency two years later. Together with the observed concurrent brain–behavior relationship of face‐selective response in the fusiform gyrus and RAN, we suggest that preliterate face processing in the ventral stream might be a promising candidate to test the neural “reading readiness” of children and should be further examined as a possible preliterate neural marker of reading acquisition.

## CONFLICT OF INTEREST

The authors declare no competing financial interests.

## AUTHOR CONTRIBUTIONS

Johanna Liebig, Eva Froehlich, Johannes C. Ziegler, and Arthur M. Jacobs designed the study; Johanna Liebig analyzed the data with advice from Eva Froehlich and Teresa Sylvester; Johanna Liebig drafted the article. All authors contributed to the final version of the article.

### ETHICS STATEMENT

The study was approved by the Ethics Committee of the German Association for Psychology (DGPs).

## INFORMED CONSENT

Parents and children gave written informed consent to all parts of the study.

## Supporting information


**Appendix** **S1.** Supporting Information

## Data Availability

Data of the study is available on request to the corresponding author.
