# Supplementary material for: Neural processing of vision and language in kindergarten is associated with prereading skills and predicts future literacy
Source: Hum Brain Mapp. 2021 May 4;42(11):3517–33. doi: 10.1002/hbm.25449 (PMC8249894; doi:10.1002/hbm.25449)
Supplement: Supplementary file 1 — Appendix S1. Supporting Information [file HBM-42-3517-s001.pdf]

Supplementary Tables

Table S1. Baseline contrasts of vision and language

Visual Processing	Cluster		Peak	MNI		
	p_{FWE}	*k*	*T*	x	y	z
**Checkerboards**						
L Calcarine	<.001	12784	23.75	-8	-90	4
R Calcarine			22.00	10	-88	-2
R Lingual			21.74	16	-74	-6
R SPL	.030	351	7.96	24	-70	44
R SPL/ AG			5.87	28	-60	52
R SPL			3.99	22	-60	60
**Houses**						
R Occipital Pole	<.001	9744	19.69	12	-94	12
L Lingual/ oFuG			19.06	-14	-82	-12
L Occipital Pole			18.51	-12	-98	12
**Faces**						
L Occipitial Pole/ Superior Occipital	<.001	7639	12.60	-14	-96	16
L IOG/ oFuG			12.11	-42	-74	-12
L IOG			11.90	-38	-82	2
**Written Words**						
L oFuG / IOG	.007	518	8.51	-26	-92	-14
L oFuG/ Occipital Pole			8.11	-16	-98	-8
L IOG			6.88	-22	-92	2
Auditory Processing						
**Spoken Words**						
L STG	< .001	1921	14.32	-60	-10	0
L STG/ PT			14.15	-58	-32	8
L STG/ PT/ MTG			13.38	-62	-24	2

Results of group-level one-sample *T*-Tests to examine fMRI stimulus conditions against null (baseline contrast). Clusters are presented with a threshold of $p < .001$ (uncorrected), $p_{FWE} < .05$. xyz = MNI coordinates of cluster center of mass, *k* = number of voxels; *T* = T-value of peak activation; R = right hemisphere, L = left hemisphere; SPL = Superior Parietal Lobe; AG = Angular Gyrus; oFuG = Occipital Fusiform Gyrus; IOG = Inferior Occipital Gyrus; STG = Superior Temporal Gyrus; PT = Planum Temporale; MTG = Middle Temporal Gyrus.

Table S2. Differential contrasts for visual stimuli

	Cluster		Peak	MNI		
	p_{FWE}	*k*	*T*	x	y	z
**Houses > [Faces, Written Words]**						
R Occipital Pole	<.001	10340	21.4	12	-92	12
L Occipital Pole			19.7	-8	-90	4
**Faces > [Houses, Written Words]**						
L Cuneus	<.001	1871	9.95	0	-82	22
R Cuneus			9.61	6	-86	26
L Calcarine/ Lingual			7.66	-2	-76	8
BL SMC	.002	670	6.44	0	-8	48
L SMC			5.95	-4	0	48
R SMC			5.72	6	2	48
**Written Words > [Houses, Faces]**						
No voxels survived						

Results of group-level one-sample *T*-Tests to examine specific response to houses, faces and written words (differential contrasts). Clusters are presented with a threshold of $p < .001$ (uncorrected), $p_{FWE} < .05$. MNI = coordinates of cluster center of mass, *k* = number of voxels; *T* = T-value of peak activation; R = right hemisphere, L = left hemisphere; BL = bilateral; SMC = Supplementary Motor Cortex.

Table S3. Region of interest analysis of brain-behavior association with rapid automatized naming.

	MNI			Cluster	Peak	
	x	y	z	k	*T*	p_{corr}
Generic Regions						
**Faces** *(positive association)*						
L Fusiform	-42	-48	-24	17	*4.17*	*<.001*
**Faces > [Houses, Words]** *(positive association)*						
R Fusiform	30	-58	-12	276	*5.79*	*<.001*
R oFuG	32	-78	-6			
R IOG	26	-88	-2			
L Fusiform	-42	-46	-20	168	*4.35*	*<.001*
L oFuG	-36	-66	-14			
L STG	-46	-46	20	10	*3.88*	*<.001*
**Written Words** *(negative association)*						
R Fusiform	32	-56	-14	78	*4.29*	*<.001*
L SMG	-58	-48	22	38	*4.28*	*<.001*
R AG	46	-44	20	10	*3.85*	*<.001*
**Written Words > [Houses, Faces]** *(negative association)*						
L STG	-56	-46	18	17	*3.98*	*< .001*

**Spoken Words**						
(negative association)						
L Fusiform	-38	-50	-10	29	*4.40*	*<.0005*
Language Specific Regions						
**Written Words**						
(negative association)						
L STG	-56	-52	20	71	*4.76*	*<.0005*
L SMG	-46	-48	22			
L Precuneus	-12	-74	40	15	*4.04*	*<.0005*
**Written Words > [Houses, Faces]**						
(negative association)						
L STG	-56	-52	20	18	*4.11*	*<.0005*
**Spoken Words**						
(negative association)						
L Precuneus	-14	-58	46	77	*4.78*	*<.0005*
L SPL	-16	-62	54			
L SPL	-30	-58	56	20	*3.78*	*<.0005*
L Fusiform	-40	-54	-16	11	*3.67*	*<.0005*

Results of independent ROI analyses on group-level with rapid automatized naming as regressor of interest at $p < .001$ (uncorrected) and $k > 10$ for baseline and differential contrasts; results were additionally controlled for the number of regression models at $p_{corr} < .0005$. Generic ROIs are bilateral fusiform gyrus, bilateral superior temporal gyrus; language specific ROIs are left inferior occipital gyrus, left ventral occipito-temporal gyrus, bilateral middle temporal gyrus, left superior parietal lobe. Significant activation extending results of the whole brain analysis are marked in light yellow. MNI = coordinates of cluster center of mass, *k* = number of voxels, *T* = T-value of peak activation, L = left hemisphere, R = right hemisphere, oFuG = occpital fusiform, IOG = inferior occpital gyrus, STG = superior temporal gyrus, SMG = supramarginal gyrus, AG = angular gyrus, SPL = superior parietal lobe.

Table S4. Region of interest analysis of brain-behavior association with reading fluency.

	MNI			Cluster	Peak	
	x	y	z	k	*T*	p_{unc}
**Faces**						
(positive association)						
L STG	-50	-20	4	15	*4.14*	*<.001*
L oFuG	-24	-76	-4	11	*3.94*	*<.001*

Results of independent ROI analyses on group-level with rapid automatized naming as regressor of interest at $p < .001$ (uncorrected) and $k > 10$. Only the baseline contrast of faces showed significant results. ROIs are bilateral fusiform gyrus and bilateral superior temporal gyrus. Significant activation extending results of the whole brain analysis are marked in light yellow. MNI = coordinates of cluster center of mass, *k* = number of voxels, *T* = T-value of peak activation, L = left hemisphere, STG = superior temporal gyrus, oFuG = occipital fusiform.

Supplementary Figures

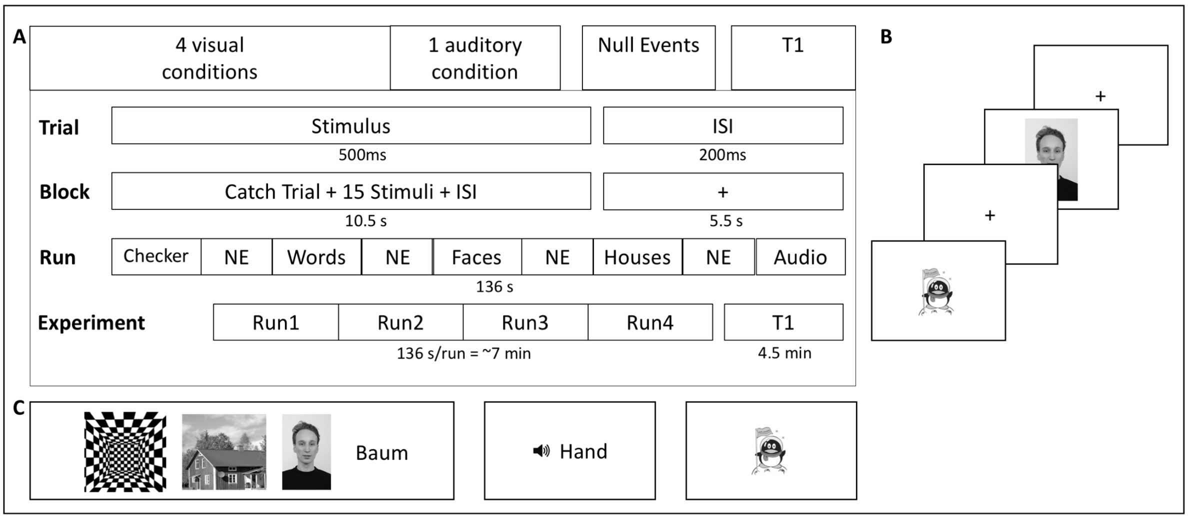

Figure S1. FMRI task design. A) experimental design is shown including information on different levels. B) Exemplary Stimulus presentation is depicted. Each block started with a catch trial (mascot of the project) followed by a fixation cross (ISI = inter-stimulus interval) that was then replaced by the first stimulus of the block. C) Examples of each stimulus condition are given (left box: visual conditions, mid: auditory condition, right box: catch trial image). NE = null-event.

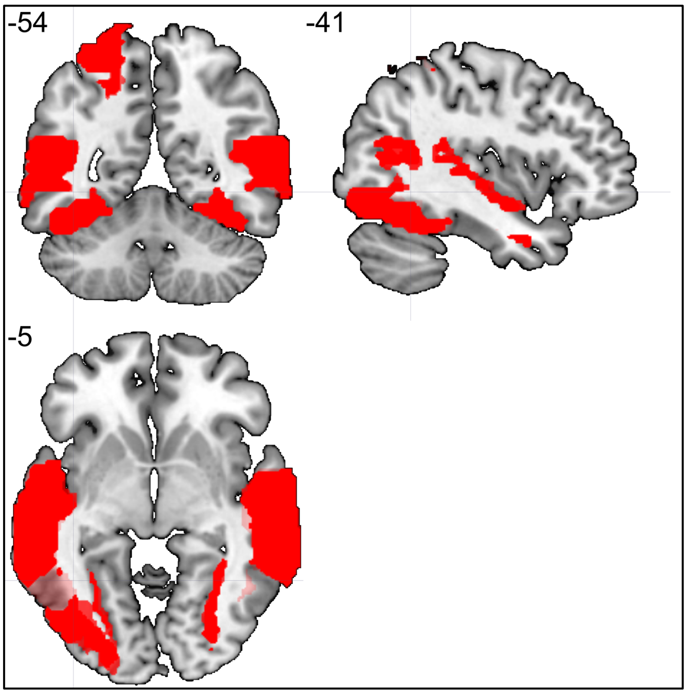

FIGURE S2. Anatomically defined regions of interest (ROI) used for the brain-behavior analysis. Exemplary slices are shown (numbers indicate coordinates).

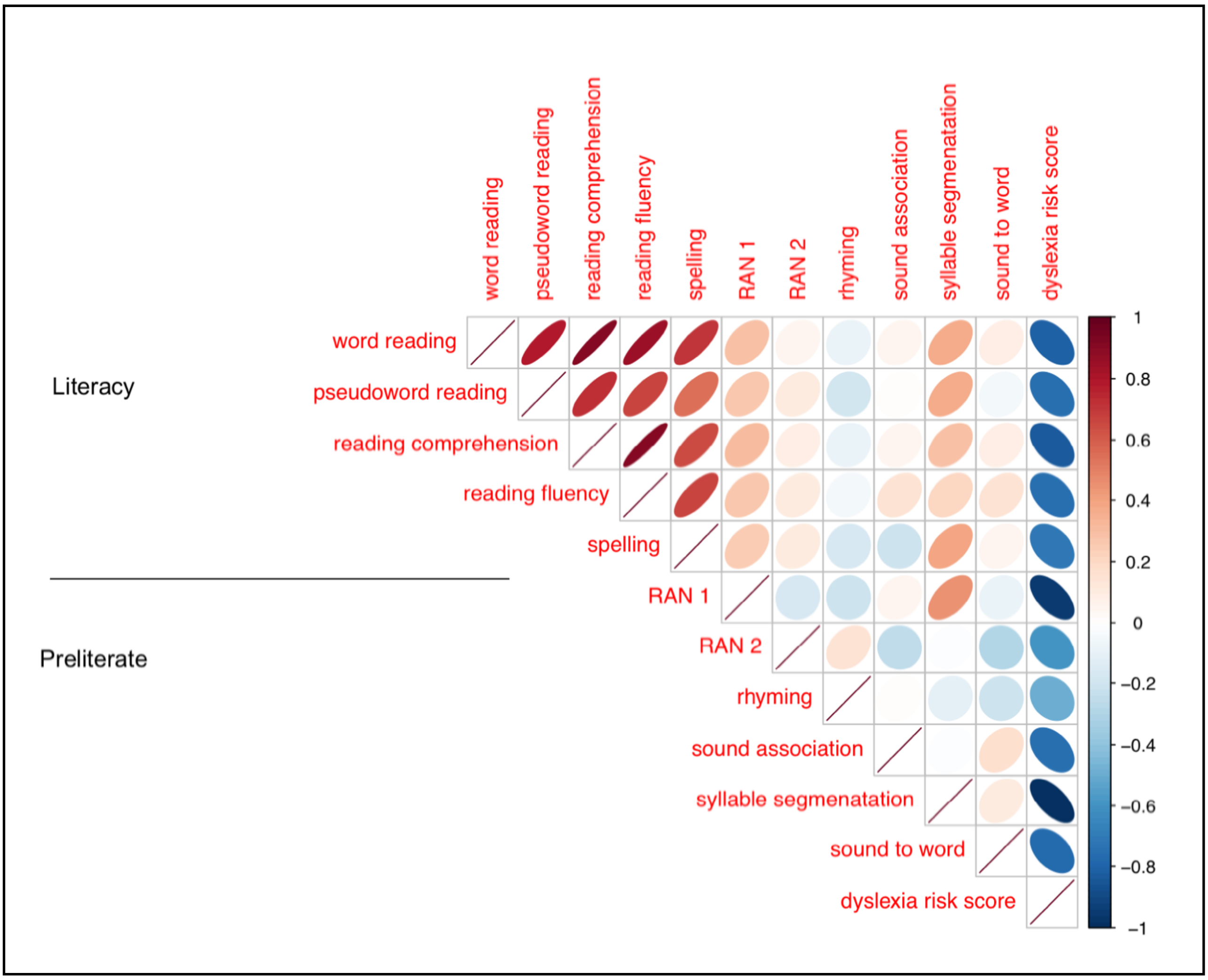

FIGURE S3. Correlation matrix of preliterate (T1) and literacy skills (T2). Red ellipses display positive, blue ellipses negative correlation. The intensity of the color indicates the strength of the relationship (see color legend). Lower values of dyslexia risk points (BISC; Jansen, 2002) indicate higher performance in prereading abilities in kindergarten. BISC: Bielefelder Screening zur Früh-erkennung von Lese-Rechtschreibschwierigkeiten, word reading: Salzbuger Lese- und Rechtschreibtest (SLRT), pseudoword reading: SLRT, reading fluency: Salzburger Lese-Screening (SLS), reading comprehension: Ein Leseverständnistest (ELFE), spelling: Deutscher Rechtschreibtest (DERET).
